# Tree growth characteristics, soil parameters, and soil organic carbon of highland *Juniper* and *Acacia* forests in Southwestern Saudi Arabia

**DOI:** 10.3389/fpls.2025.1693276

**Published:** 2026-01-30

**Authors:** Ahmed M. Abbas, Mohamed T. Ahmed, Khalid A. Ibrahim, Ahmed A. Hussain, Stephen J. Novak

**Affiliations:** 1 Department of Botany and Microbiology, Faculty of Science, Qena University, Qena, Egypt; 2 Biology Department, College of Science, King Khalid University, Abha, Saudi Arabia; 3 Prince Sultan Bin Abdelaziz for Environmental Research and Natural Resources Sustainability Center, King Khalid University, Abha, Saudi Arabia; 4 Department of Biological Sciences, Boise State University, Boise, ID, United States

**Keywords:** clay content, elevation and climate, soil bulk density, soil organic carbon content, soil organic carbon density, soil organic carbon stock, species identity

## Abstract

Variation in SOC affects the global carbon cycle and climate change and it also determines differences in soil fertility and consequently the plant communities growing in different habitats. The overall goal of this study is to determine variation in SOC in the soils associated with two trees that dominate forests in the highlands of southwestern Saudi Arabia. *Juniperus procera* dominates forests at higher elevation sites and *Acacia gerrardi* dominate forests at lower elevations in these highlands. Soils of the *J. procera* study site had significantly higher mean values for SOC content and SOC density, compared to the mean values of the *A*. *gerrardii* study site. The mean value of SOC stock at the *J*. *procera* study site was higher than that of the *A*. *gerrardii* site, but these differences were not significant. Conversely, the value of soil bulk density (SBD) at the *A*. *gerrardii* was significantly higher compared to the *J*. *procera* study site. Values of SOC content and SBD were inversely related, and this relationship appeared stronger for the *J*. *procera* site. The higher SOC values of the *J*. *procera* study site appear to be influence by the significantly larger values of DBH, tree radius, and basal area of these trees, compared to the *A*. *gerrardii* trees. Values for five of six soil parameters did not differ significantly between the two study sites, but the clay content of the *J*. *procera* study site was significantly higher than the *A. gerrardii* site. SOC variation at the two study sites appears to be influenced by differences in elevation, climate, species identity, soil bulk density, and clay content. However, the relative importance of these factors on SOC variation was not assessed. Additionally, the roles of litterfall and understory vegetation in contributing to the SOC variation of these two forest ecosystems are yet to be determined. *Juniperus procera* and *A*. *gerrardi*, and the communities they occur in, should be the focus of effective management actions because these species and communities play an important role in carbon sequestration and are also important for biodiversity conservation and the maintenance of ecosystem function.

## 1 Introduction

Human activities generate more atmospheric carbon dioxide (CO_2_) than natural processes can eliminate; thus, rapidly increasing the concentration of CO_2_ in the atmosphere. In 2024, the global average CO_2_ concentration was 422.7 ppm, a record high (Lindsey, 2025). This increase in the global CO_2_ concentration leads to climate change and extreme weather events (Trenberth et al., 2014; Williams et al., 2020; IPCC, 2021). Soil is a critical component of climate regulation either by emitting greenhouse gases or through the sequestration of organic carbon (OC), which reduces the concentration of CO_2_ in the atmosphere (McBratney et al., 2014; Devi, 2021; Beillouin et al., 2022, 2023). Globally, the uppermost layers of soil (2–3 m depth) contain between 1, 500-2, 400 Pg OC, which is approximately two to three times the amount of carbon in the atmosphere (~800 Pg C) and three to four times the amount of carbon in vegetation (~500 Pg C) (FAO, 2015; Devi, 2021; Beillouin et al., 2022). Alteration of soil organic carbon (SOC) concentrations can affect the Earth’s global carbon cycle (Walter et al., 2016; Devi, 2021), and efforts to increase SOC have been proposed as natural climate solutions (Beillouin et al., 2023).

Variation in SOC not only affects the global carbon cycle and climate change; it also has an impact on soil fertility (the nutrient supply of soils) and consequently the plant communities growing in different habitats, biodiversity patterns, and food production (Beillouin et al., 2023; Yao et al., 2023). High SOC levels supply the optimal conditions for plant growth, promotes soil nutrient cycling, and influences the structure and function of ecosystems (Yang et al., 2022; Yao et al., 2023). The amount and distribution of SOC across the globe is influenced by many interacting biotic and abiotic conditions.

Higher levels of SOC are associated with the soils of temperate regions compared to arid regions (Beillouin et al., 2021; 2022), higher elevation and north-facing slopes compared to lower elevation and south-facing slopes (Hontoria et al., 1999), forest communities compared to grassland and desert communities (Díaz-Pinés et al., 2011; Angst et al., 2019; Devi, 2021; Kuznetsova, 2022; Yao et al., 2023), plant species with higher biomass accumulation and litter fall compared to lower biomass accumulation and litter fall (Devi, 2021; Kuznetsova, 2022), and diverse soil microbial communities compared to those with lower diversity (2024). For example, forests comprise 31% of the land area across the globe (Yang et al., 2022), yet forest soils store approximately 70% of the Earth’s SOC (Pan et al., 2011).

In addition, higher levels of SOC are associated with soils that have relatively high fine soil particles like clay, higher soil moisture, and lower soil bulk density, compared to soils with coarse particles such as sand (Hassink, 1997; Boudjabi and Chenchouni, 2022; Chenchouni and Neffar, 2022; Osman et al., 2023). Variation in SOC is also influenced by anthropogenic activities that lead to climate change, and land-use, or land-cover, changes such as deforestation, afforestation, woody-plant encroachment, and agricultural development (Jackson et al., 2002; Albaladejo et al., 2013; Mureva et al., 2018; Buraka et al., 2022; Yang et al., 2022).

Despite abundant SOC studies that have been published (Ayoub and Niazi, 2001; Beillouin et al., 2021, 2022, 2023), data gaps still exist (Beillouin et al., 2021). For instance, the vast number of published SOC studies were conducted in just five countries, in regions with mostly temperate climates: United States of America, China, Brazil, Australia and India (Beillouin et al., 2022). Thus, there is an urgent need to conduct such studies in arid and semi-arid habitats, and in other countries and regions around the globe. There is also a need for additional SOC studies to facilitate the mitigation of ongoing climate change, assess the consequences of the extensive land-use change currently taking place, and to maintain soil health and agricultural productivity (Beillouin et al., 2022, 2023).

One such region with an arid and semi-arid climate in need of more SOC studies is the Arabian Peninsula, and specifically Saudi Arabia. Such studies are especially warranted because of the severe and adverse impacts of climate change predicted for Saudi Arabia (El-Rawy et al., 2023). To the best of our knowledge, no SOC studies have been published based on the analysis of soils in high elevation forests of southwestern Saudi Arabia, which experience arid and semi-arid climate conditions. Such studies are needed because forests and meadow in this region contain higher plant species diversity and productivity compared to other regions in Saudi Arabia, and these forests have historically experience overexploitation and extensive timber harvesting (Zohary, 1973; Abulfatih, 1979, 1984; Miller et al., 1996; Hegazy et al., 1998; El-Juhany and Aref, 2013; Al-Namazi et al., 2022; Sayed and Masrahi, 2023).

Beginning with the Cenozoic Era (~65 million years ago), collision of the Arabian and African tectonic plates caused uplifts that created the Asir Mountain Range in the Asir region of southwestern Saudi Arabia (Abulfatih, 1984; Miller et al., 1996; Almalki et al., 2022). These events resulted in Jabal Soudah Peak, in the Soudah Mountains, which is the highest peak in Saudi Arabia, at 3, 015 m above sea level (a.s.l.) (Miller et al., 1996; Almalki et al., 2022). This mountainous region consists of a fragmented landscape with climatic diversity, which leads to high plant species diversity (Abulfatih, 1979, 1984; Hegazy et al., 1998; El-Juhany and Aref, 2013; Al-Namazi et al., 2022). In Saudi Arabia, the vegetation of the southern and southwestern highlands represents the northern limit of the Afromontane Flora: 44% of the flora in these two regions consist of Sudanese elements, characteristic of the savanna scrub and montane woodlands elevation zones (Zohary, 1973), which also occurs in Somalia, Ethiopia, and Kenya. The dominant tree species in the mountains of southwestern Saudi Arabia include *Juniperus procera* Hochst. Ex. Endl. (Cupressaceae), *Acacia* spp. L. (Fabaceae), *Olea europaea* L. (Oleaceae), and *Pistacia atlántica* Desf. (Anacardiaceae) (Abulfatih, 1979, 1984; Hegazy et al., 1998; Almalki et al., 2022). For centuries, residents of treeless areas in the Asir region continuously reduced the size of these highland forests through timber harvesting to obtain wood for fuel and construction material, and through controlled burns to create high-elevation pasture lands (Brooks and Mandil, 1983). Evidence suggests that such activities shifted these woodlands to the north and west, significantly hindering the regeneration of tree species in this mountainous region.

The overall goal of this study is to determine variation in SOC in the soils associated with two trees that dominate forests in the highlands of southwestern Saudi Arabia, *J*. *procera* and *Acacia gerrardi* (Benth.). This goal will be achieved by answering three specific questions: 1) how do the values of SOC, to a depth of 40 cm, compare for our *J*. *procera* and *A*. *gerrardi* study sites? 2) what is the relationship between canopy size and volume and SOC variation at our two study sites?, and 3) how does soil particle size distribution, soil chemistry, and soil bulk density (SBD) in stands of these two trees compare and how are soil parameters and SOC variation related? Based on the background information provided here, we hypothesize that soils of the forest stand with higher vegetation cover (i.e., canopy size and canopy volume) will have higher SOC values compared to the forest stand with lower vegetation cover. Additionally, we hypothesize that higher SOC values will be associated with soils with higher clay content compared to sandy soils and lower SBD compared to higher SBD.

## 2 Materials and methods

### 2.1 Study species

*Juniperus procera*, known as African juniper, East African juniper, African pencil-cedar or “Arar” in Arabic, occurs across mountainous regions in East Africa and the Arabian Peninsula. Kerfoot (1975) proposed that the evolutionary origin of *J*. *procera* took place in the mountains of Saudi Arabia, with the species subsequently migrating southward to mountainous regions in East Africa. It is a mid-sized tree, typically 20–25 m tall and rarely reaching 40 m in height, with a trunk up to 1.5–2 m diameter and a conical, rounded, or irregular crown. This evergreen, predominantly dioecious tree is wind-pollinated and occurs naturally in highlands ranging from 1000 to 3500 m a.s.l. and occurs in habitats in which the temperature ranges from 5°C to 20°C (Chaudhary, 1999; Farjon, 2005; El-Juhany, 2015). It is listed as a threatened species on the IUCN Red List due to overexploitation (Farjon, 2013). However, in the mountains of southwestern Saudi Arabia, *J*. *procera* is one of the dominant tree species at an elevation range of between 2000 and 3000 m.a.s.l., with decreasing abundance at lower elevations (El-Juhany and Aref, 2013; El-Juhany, 2015). This tree species is an important component of ecosystem dynamics in this region, enhancing biodiversity, reducing soil erosion, conserving water, and providing shade to wildlife and livestock (Abulfatih et al., 1989; Aref et al., 2003).

*Acacia gerrardii* (common names, Gerrard’s wattle, red thorn, and grey-haired acacia) is native to subtropical regions of Africa and western Asia (Dharani, 2006). This species is one of the most common acacias in the Arabian Peninsula, particularly in Saudi Arabia (Aref et al., 2003; Hosny et al., 2018). *Acacia gerrardii* is a small evergreen tree, reaching 5–10 m tall, with a rounded crown that is usually larger in diameter than it is in height, and provides shade to wildlife and livestock (Elias, 1981). The species has a deep taproot and occurs in arid and semi-arid savannahs and open woodlands in well-drained soil and can tolerate a broad range of environmental conditions, which makes it well-suited to cope with drought stress (Aref et al., 1995). *Acacia gerrardi* forms symbiotic relationships with nitrogen-fixing *Rhizobium* bacteria and mycorrhizal fungi (Shetta and Al-Shahrani, 2014; Hashem et al., 2016), which enhances the ecological importance of this species. The tree provides habitat and food for various wildlife species, including insects, birds, and mammals, and its flowers attract pollinators, contributing to local biodiversity. *Acacia gerrardii* holds considerable promise for sustainable development (Bethlenfalvay et al., 1992). Additionally, this species is planted to reduce soil erosion and provides firewood, fiber, food, fuel, tannins, and gum, and they are used in handicrafts, ornamental purposes, and agroforestry systems (Alamgir and Hossain, 2005; Abbaker Ibrahim Yousif and Rong Wang, 2021).

### 2.2 Study sites

**Figure 1:**
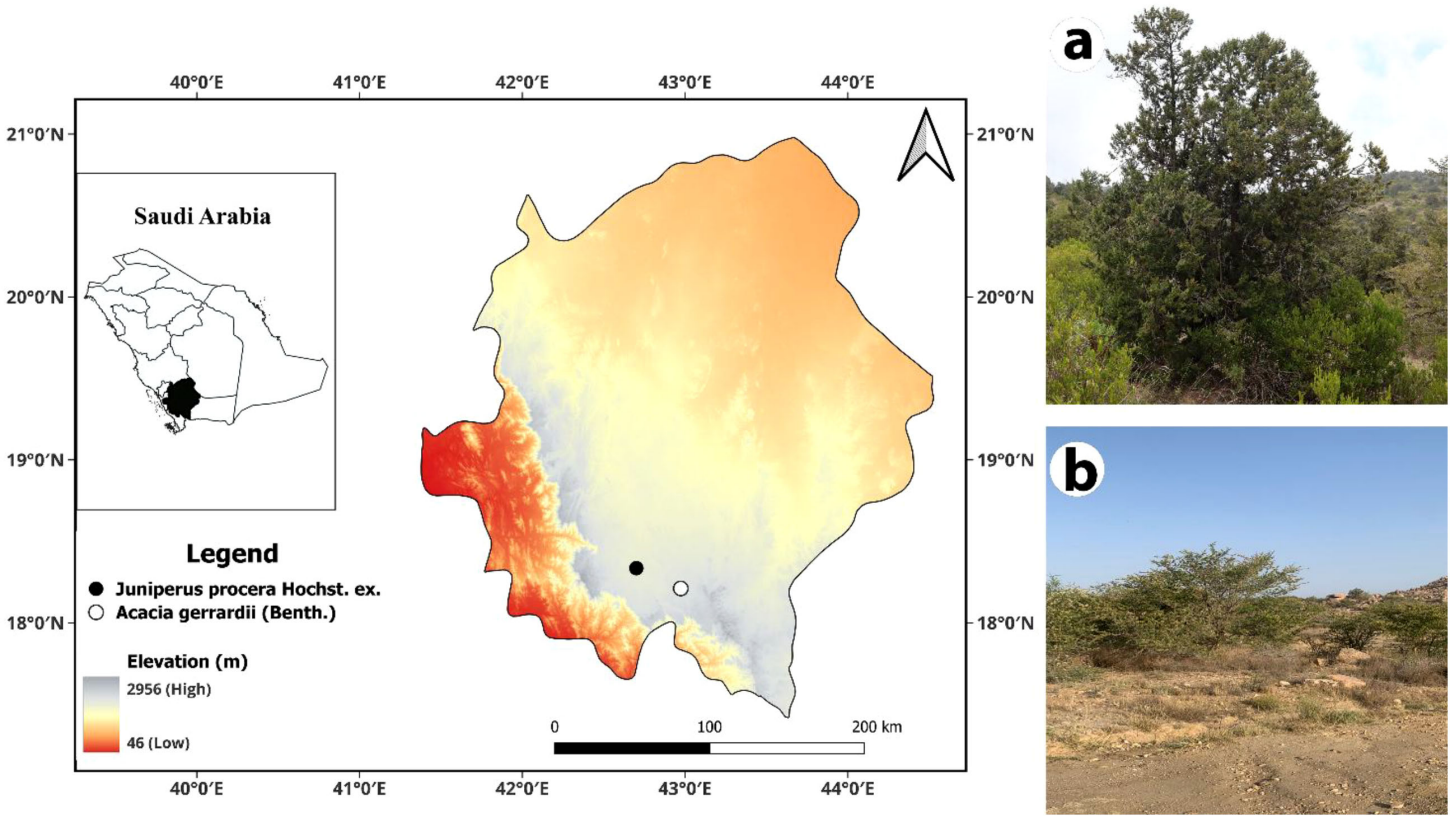
Map showing the locations of the *Juniperus procera* and *Acacia gerrardii* study sites in Asir Province in southwestern Saudia Arabia. Elevations are color coded in the map insert of the Asir Province. **(a)** Photo of a representative *Juniperus procera* sampling location. **(b)** Photo of a representative *Acacia gerrardii* sampling location.

To conduct this research, we set up two forested study sites in the Asir region of Southwestern Saudi Arabia, one in a *J*. *procera* dominated area and the other in plant communities dominated by *A*. *gerrardi* (**Figure 1**). These two study sites are in the Precambrian Shield (or the Arabian Shield) geologic unit (Pollastro et al., 1999; Almalki et al., 2022), and are characterized by Kastanozem-Leptosol soils with either silt loam (the *J*. *procera* study site) or sandy loam soils (the *A. gerrardi* study site) (Fischer et al., 2008; Almalki et al., 2022). Mean soil depths in this region range from 0–3 m in depth, with the soils at the two study sites generally shallower than 3 m and quite gravelly. For a more detailed description of the soil taxonomy of the two study areas refer to the Ministry of Agriculture and Water, 1986.

The *J*. *procera* study site was located near Jabal Soudah Peak (18° 20 ′ 05′′ N, 42° 42 ′ 02′′ E), approximately 25 km east of Abha, Saudi Arabia (**Figure 1**). Six sampling locations were established at this study site, and the elevations of these six sampling locations ranged from 2755 to 2790 m a.s.l (mean = 2775.5 m). Six sampling locations were set up at this study site because this number effectively sampled the variability we observed in this forest type, which led to a sampling intensity of approximately 1.5% (data not shown). The climate of these sampling locations varies based on the season of the year, elevation, and aspect. The Soudah Mountains experience fluctuating precipitation levels due to a monsoonal flow, which comes from the southwest (i.e., the Red Sea). This monsoonal flow delivers moist oceanic winds to this mountainous region. As these winds ascend this mountainous region, they often result in thunderstorms, especially during the summer, with most precipitation occurring in April/May and July/August. Annual precipitation averages 600 to 800 mm in the Soudah Mountains, exceeding 1, 000 mm in the wettest areas. High plateau areas located at lower elevations receive 300 to 500 mm of precipitation, sharply declining to below 100 mm in the eastern lowlands (The General Authority of Meteorology and Environmental Protection website; https://www.mewa.gov.sa/en/partners/pages). Summer temperatures in the highlands can reach 20 to 25°C, while winter averages can reach 10°C, with frost possible at higher elevations and occasional snowfall on Jabal Soudah Peak (Miller, 1994). The mountainous region of southwestern Saudi Arabia features a semi-arid climate and is conducive to the growth of non-xerophytic tree and shrub species. This entire area is recognized for its rich species diversity and high levels of endemism, despite the presence of ancient human settlements in these higher elevations (NCWCD, 2005).

The *A. gerrardi* study site was located near Al Runah (18° 12′ 37′′ N, 42° 85′ 24′′ E), approximately 45 km east of Abha, Saudi Arabia (**Figure 1**). Six sampling locations were established at this study site, and the elevations of these six sampling locations ranged between 2190 and 2269 m a.s.l., with a mean of 2235.7 m. These locations occurred in and near wadi Bisha. Six sampling locations were set up at this study site because this number effectively sampled the variability we observed in this forest type, which led to a sampling intensity of approximately 2% (data not shown). Al Runah experiences a subtropical highland climate. Summer temperatures can reach 40°C, with winter temperatures ranging between 15 and 25°C. Average annual precipitation is approximately 100 mm/year, with most of this precipitation falling between November and April (The General Authority of Meteorology and Environmental Protection website: https://www.mewa.gov.sa/en/partners/pages).

### 2.3 Sampling of tree growth characteristics

A microplot with an area of 625 m² (25 m × 25 m) was deployed in each of the six sampling locations established at the *J*. *procera* or *A*. *gerrardii*. study sites. A stratified random sampling technique was employed, which has been shown to be effective for use in non-homogeneous forest stands (Shiver and Bruce, 1996). All trees in each microplot were counted. We measured eight tree growth characteristics: diameter at breast height (DBH, measured 1.4 m above ground level), crown depth, crown diameter, crown volume, tree height, tree radius, basal area, and crown-to-height ratio for all trees. Trees were classified into DBH categories ranging from 0–5 cm to > 45 cm, along with tree radius and basal area measurements for each tree as described by Abbas et al. (2021). To calculate the crown-to-height ratio (C/H ratio), we divided the crown volume by the tree height. Crown diameter was measured by projecting the crown edges onto the ground and measuring the distance along an axis connecting these edges. This diameter can be used to estimate the crown area, which is used in the crown surface area and volume calculations by averaging two axes (West, 2015). In this study, we averaged the diameters of two perpendicular axes (N–S and E–W). Crown depth was determined as the vertical distance from the top of the tree to the crown base, identified by the lowest complete whorl of branches or the lowest branch that contributes to the canopy. We expressed crown depth as the crown length ratio, calculated by dividing the crown length by the tree height. Crown volume (Cv) was estimated using the crown diameter (D) and crown depth (L), based on the formula provided by West (2015).

A scatter matrix diagram was prepared to determine the correlation coefficients of elevation with *Juniper procera* and *Acacia gerardias* tree growth characteristics, as well as the correlation between tree growth characteristics, at the study sites in southwestern Saudi Arabia.

### 2.4 Soil parameters

Three of the six sampling locations at the *J*. *procera* or *A*. *gerrardii*. study sites were randomly selected for soil sampling (i.e., there were three locations per tree species). At each sampling location, three soil cores were taken at a depth of 0–20 cm and the three cores were combined to form a single composite sample for analysis. The samples were air-dried and grounded. We assessed the soil particle size distribution using the sieve method, as described by Gee and Bauder (1986), allowing us to quantify the proportions of sand, silt, and clay, following sieving. To measure the soil’s pH, electrical conductivity (EC), and total dissolved solids (TDS), a portable conductivity meter (Hanna Instruments, HI 98130, Woonsocket, Rhode Island, USA) was employed. Measurements were conducted using a 1:5 soil-to-water extract solution (Allen, 1989).

### 2.5 Soil bulk density and soil organic carbon estimates

At each of the six randomly selected sampling locations described above (three locations per tree species), three soil samples were obtained to estimate soil bulk density (SBD), soil organic carbon (SOC) content, SOC density, and SOC stock. To acquire representative soil samples, the position of the three samples was chosen randomly under the tree canopies at each sampling location. Soil cores were obtained using a hand-held stainless-steel corer, which measured 100 cm in length and had a 70 mm diameter. The coring device was inserted into the soil up to a depth of 40 cm. After extracting each core, it was subdivided into eight separate segments, each consisting of 5 cm depth (0–5 cm, 5–10 cm, 10–15 cm, 15–20 cm, 20–25 cm, 25–30 cm, 30–35 cm, and 35–40 cm). Each sample was then placed in sealed plastic containers and stored on ice to prevent the loss of volatile compounds and microbial activity before analyses could be conducted.

Our goal was to obtain soil samples from three sampling locations for each tree species, obtain three cores per location, obtain eight soil segments per core, for a total of 72 soil samples (segments) per species. For *A*. *gerrardii* we achieved this goal, but due to the intrusion of roots and high stone content, only 67 soil samples were obtained for the *J*. *procera* study site. Thus, we analyzed a total of 139 soil samples.

Each sample was oven-dried at 105°C for three days, cooled in a desiccator, and weighed to calculate SBD as described by Wilke (2005):


$$\rho_{sj}=\frac{m_{j}}{v_{j}}$$

Where *ρ_sj_* is SBD [g cm^−3^] of the *j*^th^ layer, *m_j_* is the mass of the soil sample [g] of the jth layer, and vj is the volume of the soil sample [cm^3^] of the *j*^th^ layer. The dried samples were ground and passed through a 2 mm mesh sieve. Soil organic carbon (SOC) content was determined by assessing soil organic matter (SOM) using the loss on ignition method at 550°C for two hours, as described by Jones (2001): SOM content [g C kg^−1^] = 1000 × (weight of oven dried sample [g] − weight of sample after ignition [g])/weight of oven dried sample [g]); SOC [g C kg^−1^] = 0.5 × SOM [g C kg^−1^] (Pribyl, 2010).

SOC density [kg C m^−3^] was estimated following the method of Han et al. (2010): *SOCdj* = *ρsj* × *SOCj*; where *SOCd_j_* is the SOC density [kg C m^−3^] of the *j*^th^ layer, *ρsj* is the SBD [g cm^−3^] of the *j*^th^ layer, *SOC_j_* is the SOC content [g C kg^−1^] of the *j*^th^ layer. SOC stock [kg C m^−2^] of a profile, expressed as a mass per unit surface area to a fixed depth, was calculated as follows (Meersmans et al., 2008):


$$SOC_{p}=\frac{\sum_{j=1}^{k}SOC_{dj}\times T_{j}}{\sum_{j=1}^{k}T_{j}}\times D_{r}$$

Where *SOC_p_* is the SOC stock [kg C m^−2^], *D_r_* is the reference depth (= 0.5 m), *T_j_* is the thickness [m] of the *j^th^* layer, and k is the number of layers.

### 2.6 Data analysis

Parametric statistical tests were performed on key variables after checking for normality and equality of variance. T-tests were used to analyze significance statistical differences between the parameters used to characterize the *J. procera* and *A. gerradii* study sites. A two-way ANOVA was performed to detect significant differences in SBD, SOC content, and SOC density between the *J. procera* and *A. gerrardii* study sites, across the eight soil depths. Mean differences among these depths were analyzed using Tukey’s range test at a significance level of *p* < 0.05. A non-linear regression analysis examined the relationship between SBD and SOC content (Eid and Shaltout, 2016). Additionally, the Student’s t-test was utilized to assess significant differences in the overall means of SBD, SOC content, SOC density, and SOC stock between the *J*. procera and A. *gerrardii* study sites, as well as variances in tree growth characteristics and soil characteristics at the two study sites. All statistical analyses were performed using SPSS 15.0 (SPSS, 2006).

We used canonical correspondence analysis (CCA) to extract gradients that explain the relation between SBD, SOC content, SOC density, and SOC stock and the six soil parameters we measured. Canonical correspondence analysis was carried out with CANOCO 5.0 for Windows (ter Braak and Šmilauer, 2012).

## 3 Results

### 3.1 Variation in tree growth characteristics

**Figure 2:**
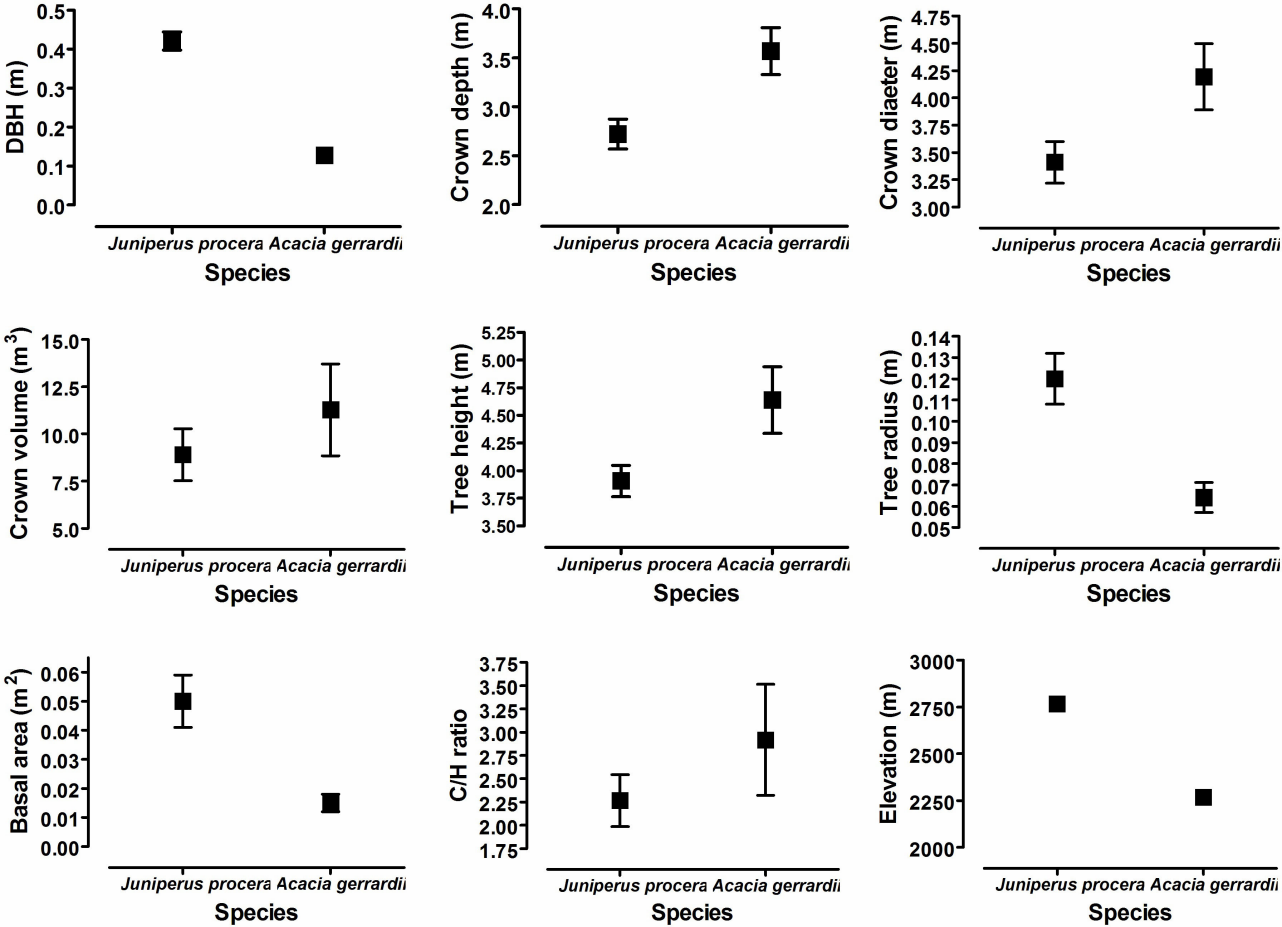
Mean values of eight tree growth characteristic measured in this study. These parameters included diameter at breast height (DBH) (m), crown depth (m), crown diameter (m), crown volume (m^3^), tree height (m), tree radius (m), basal area (m^2^), and crown volume/tree height ratio (C/H ratio) and elevation (m) for the *Juniperus procera* and *Acacia gerrardii* study sites in southwestern Saudi Arabia. Statistical analyses of these data are provided in the text.

The mean elevation of the six J. *procera* sampling locations (2775.5 m) and the six *A. gerrardii* sampling locations (2235.7 m) showed statistically significant differences (P = 0.0001) (**Figure 2**). *Juniperus procera* trees exhibited significantly higher values for DBH (*t*-test 6.745; *P* = 0.0001), tree radius (*t*-test 6.720; *P* = 0.0001), and basal area (*t*-test 5.877; *P* = 0.0001) in comparison to *A. gerrardii* trees. In contrast, *A. gerrardii* trees had significantly greater values for crown depth (*t*-test -2.886; *P* = 0.005), crown diameter (*t*-test -2.625; *P* = 0.011), and tree height (*t*-test -2.463; *P* = 0.016). Crown volume and C/H ratio did not differ significantly for the two species at the two study sites (**Figure 2**).

Correlation coefficients of elevation with all eight tree growth characteristics for *J*. *procera* and *A*. *gerrardii*, as well as the correlation between the tree characteristics, are depicted in a scatter matrix diagram (**Appendix 1**). These results showed that DBH, tree radius, and basal area are significantly associated with elevation (*r* = 0.65 and 0.64, and 0.58 respectively). Crown depth and C/H ratio were weakly, and significantly, correlated with elevation; while elevation was not significantly correlated with crown diameter, crown volume, and tress height (**Appendix 1**). The correlation coefficients between the eight tree growth characteristics display a variety of significant and non-significant relationships. For instance, DBH, tree radius, and basal area are correlated with elevation, and all three characteristics are highly correlated with each other. Crown depth is highly correlated with crown diameter and tree height and crown diameter is highly correlated with tree height (**Appendix 1**).

### 3.2 Soil parameters

**Figure 3:**
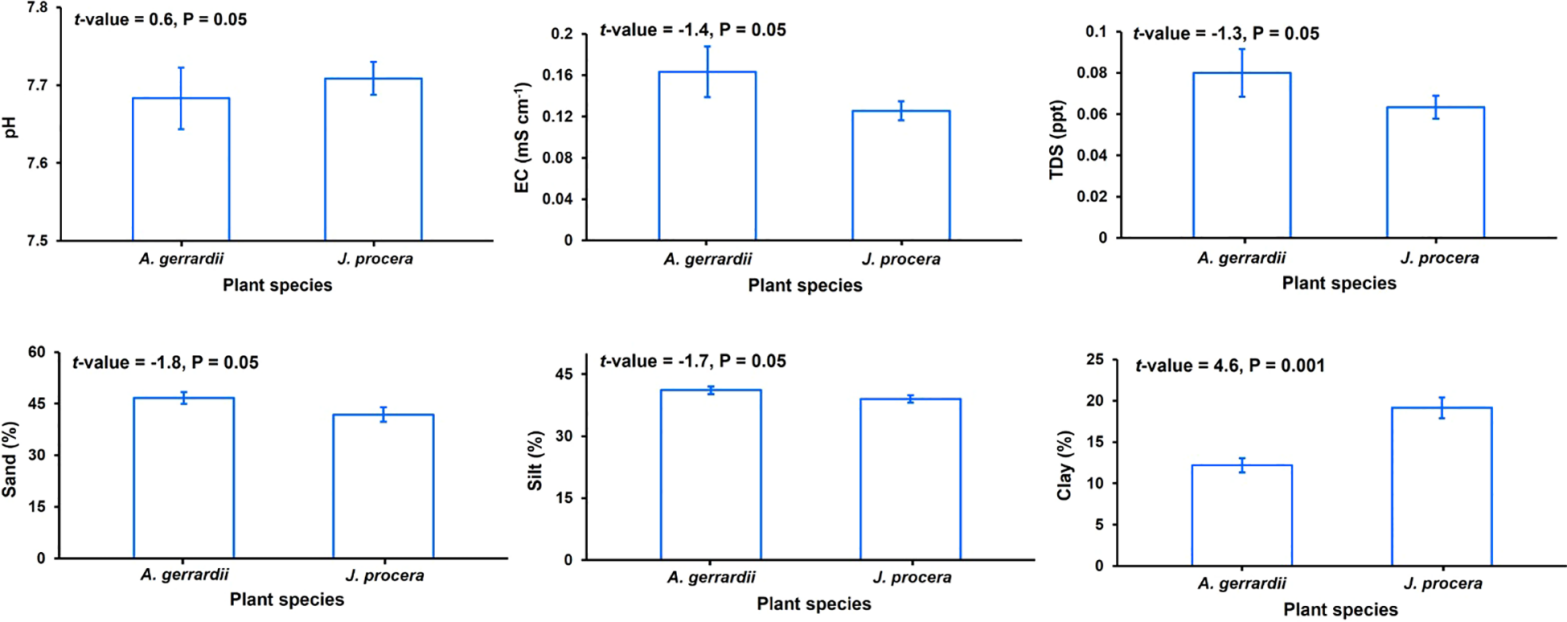
Mean values for the six soil parameters measured in this study, pH, electrical conductivity (EC), total dissolved solids (TDS), sand content, silt content, and clay content for the *Acacia gerrardii* and *Juniperus procera* study sites in southwestern Saudi Arabia. Vertical bars indicate the standard errors of the means; *t*-values indicate the results of Student *t*-tests.

Values of the soil parameters pH, EC, TDS, sand content, and silt content did not differ significantly between the *J. procera* and *A. gerrardii* study sites (*P* = 0.05, **Figure 3**). However, the clay content of the *J*. *procera* study site (19%) was significantly higher than that of the *A. gerrardii* site (13%) (*P* = 0.001).

### 3.3 Soil bulk density and soil organic carbon estimates

Soils of the *J. procera* study site had the highest mean values for SOC content and SOC density (69.2 ± 2.2 g C kg^−1^; 107.4 ± 4.5 kg C m^−3^; respectively), compared to the mean values of the *A. gerrardii* study site (32.0 ± 2.2 g C kg^−1^; 70.2 ± 5.2 kg C m^−3^; respectively), and these differences were statistically significant (*P* = 0.001) (**Table 1**). Conversely, soils at the *J. procera* study site had a significantly lower value of SBD than the *A. gerrardii* study site (1.62 ± 0.07 g cm^−3^ and 2.30 ± 5.2 g cm^−3^, respectively) (*P* = 0.001). SOC stock values at the study sites for both tree species were not statistically different (**Table 1**).

**Table 1:** Mean ± standard error of soil bulk density (SBD), soil organic carbon (SOC) content, soil organic carbon (SOC) density, and soil organic carbon (SOC) stock at the *Acacia gerrardii* and *Juniperus procera* study sites in southwestern Saudi Arabia; *t*-values represent Student’s *t*-test.

Plant species	SBD [g cm^−3^]	SOC content [g C kg^−1^]	SOC density [kg C m^−3^]	SOC stock [kg C m^−2^]
*A. gerrardii* (*n* = 72)	2.30 ± 0.10	32.0 ± 2.2	70.2 ± 5.2	28.1 ± 5.3
*J. procera* (*n* = 67)	1.62 ± 0.07	69.2 ± 2.2	107.4 ± 4.5	40.0 ± 4.2
Mean (*n* = 139)	1.97 ± 0.07	49.9 ± 2.2	88.1 ± 3.8	34.0 ± 3.6
*t-*value	5.7^***^	−12.0^***^	−5.4^***^	−1.8^ns^
Degrees of freedom	128.5	137	137	16

*ns*, not significant (i.e., *P* ˃ 0.05), ***P = 0.001.

**Figure 4:**
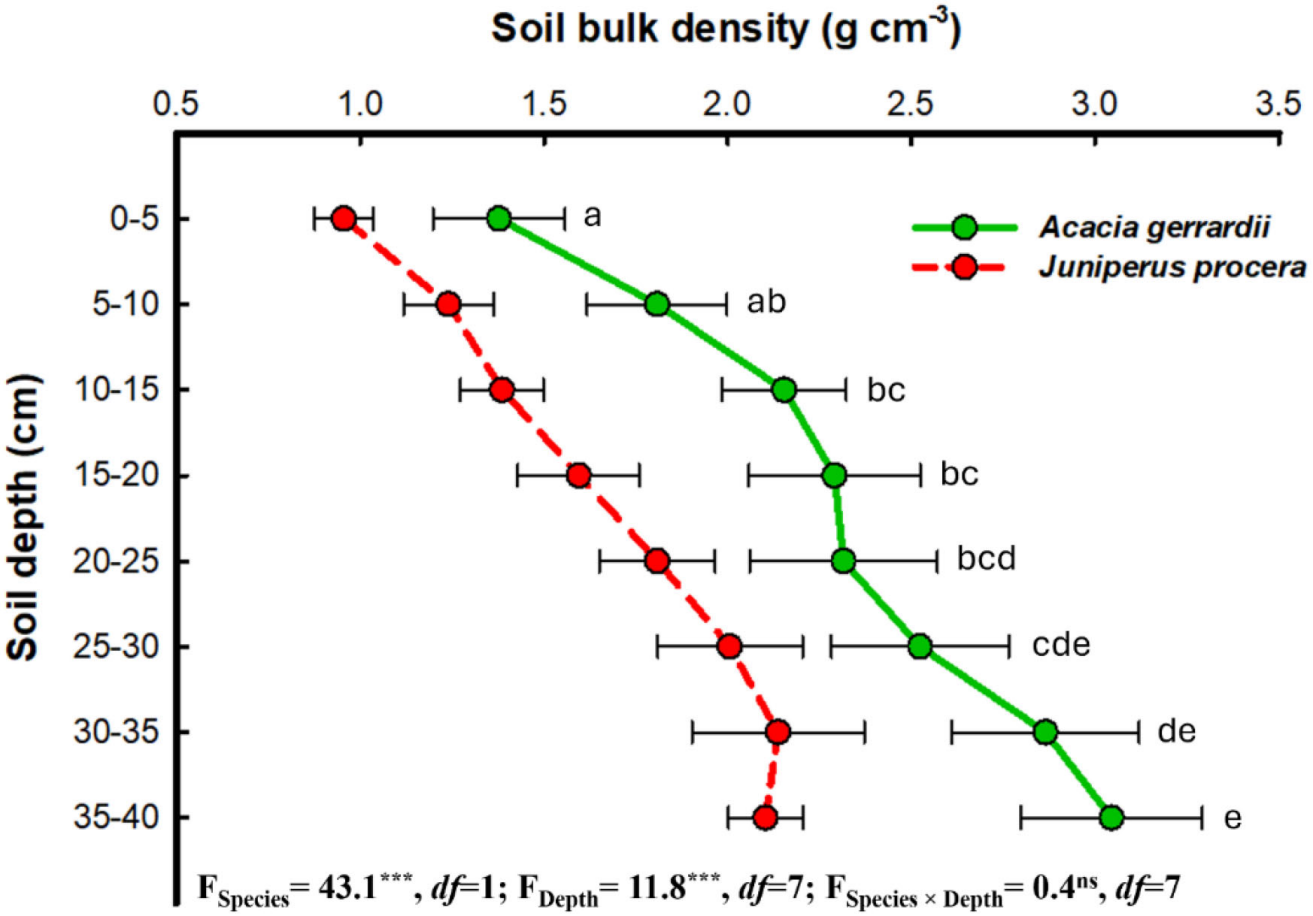
Mean soil bulk density (g cm−3) values at eight soil depths (cm) for the *Acacia gerrardii* and *Juniperus procera* study sites in southwestern Saudi Arabia. Horizontal bars indicate the standard errors of the means. *F*-values represent the two-way ANOVAs. Depths: 0 – 5, 5 –10, 10 –15, 15 – 20, 20 – 25, 25 – 30, 30 – 35, 35 – 40 cm. ***P = 0.001, *ns*: not significant (i.e., *P* > 0.05), *df*: degrees of freedom. Mean values followed by different letters are significantly different at *P* = 0.05 according to Tukey’s range test.

**Figure 5:**
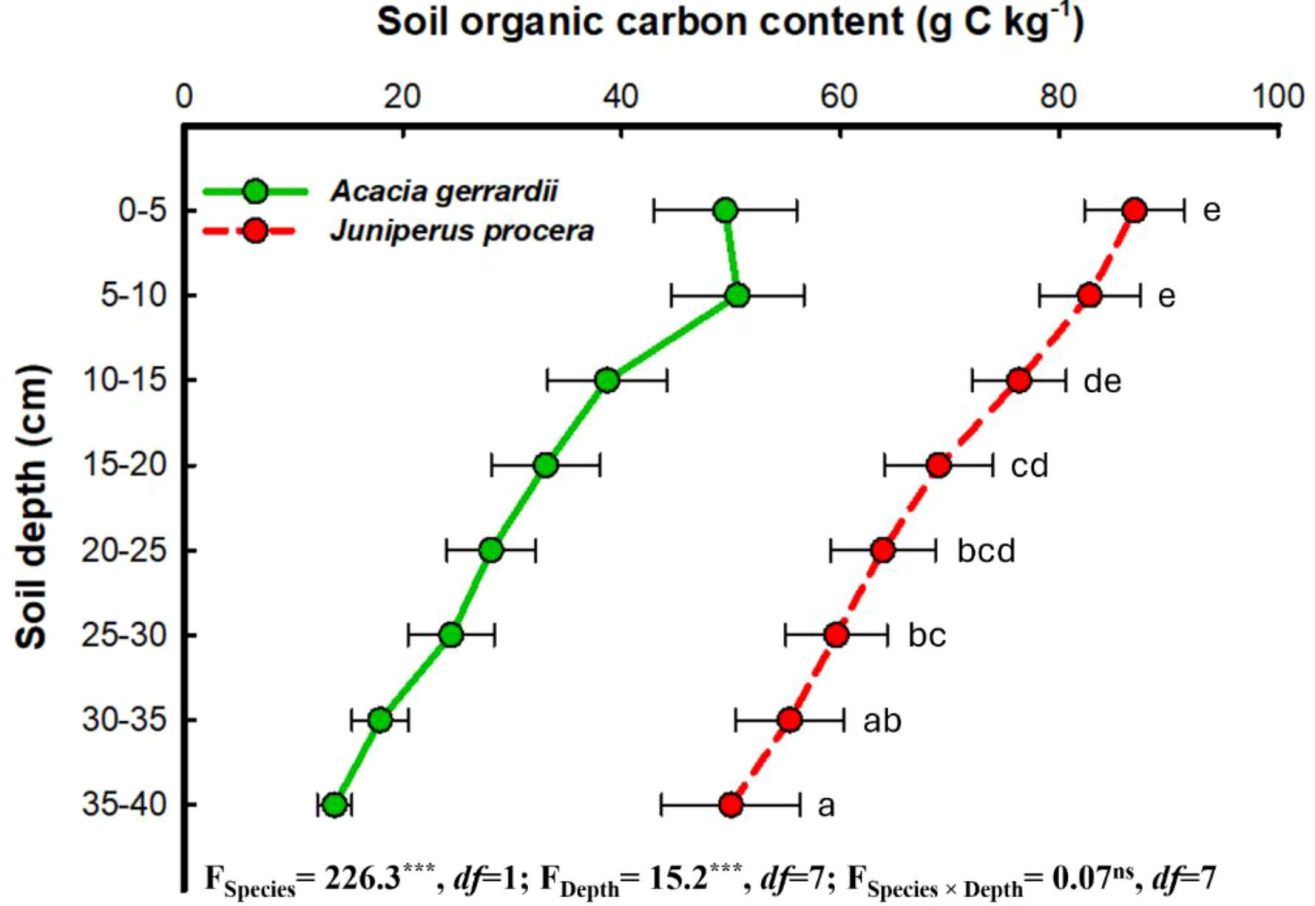
Mean soil organic carbon content (g C kg^−1^) values at eight soil depths (cm) for the *Acacia gerrardii* and *Juniperus procera* study sites in southwestern Saudi Arabia. Horizontal bars indicate the standard errors of the means. *F*-values represent the two-way ANOVAs. Depths: 0 – 5, 5 –10, 10 –15, 15 – 20, 20 – 25, 25 – 30, 30 – 35, 35 – 40 cm. ***P = 0.001, *ns*: not significant (i.e., *P* > 0.05), *df*: degrees of freedom. Mean values followed by different letters are significantly different at *P* = 0.05 according to Tukey’s range test.

The distribution of SBD significantly increased with soil depth at both the *J. procera* and the *A. gerrardii* study sites (**Figure 4**). At the *A*. *gerrardii* study site, SBD increased from 1.38 g cm^−3^ at the 0–5 cm depth to 3.05 g cm^−3^ at the 35–40 cm depth. Similarly, at the *J*. *procera* study site, values of SBD increased from 0.96 g cm^−3^ at 0–5 cm to 2.10 g cm^−3^ at 35–40 cm. In contrast, at the *J*. *procera* study site, SOC content decreased from 86.90 g C kg^−1^ at the surface to 49.99 g C kg^−1^ at the 35–40 cm depth (**Figure 5**). Values of SOC content at the *A*. *gerrardii* study site also decreased with soil depth. For the *J. procera* study site, SOC density was lowest at the upper soil levels (e.g., 82.92 kg C m^−3^ at a depth of 0–5 cm) and increased to 118.9 kg C m^−3^ at a depth of 30–35 cm (**Appendix 2**). At the *A*. *gerrardi* study site, SOC density was highest at a depth of 5–10 cm (96.10 kg C m^−3^) and decreased to 43.25 kg C m^−3^ at a depth of 35–40 cm.

**Figure 6:**
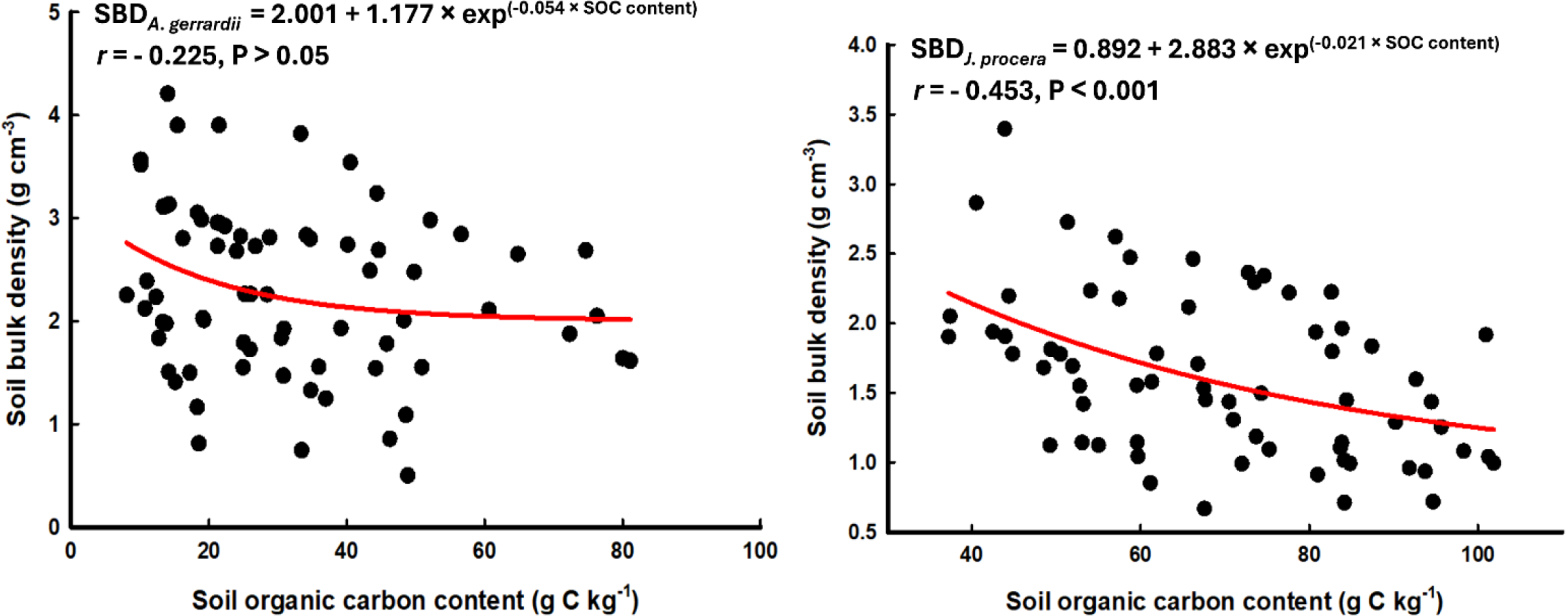
Non-linear correlation between soil organic carbon content (g C kg^−1^) and soil bulk density (g cm^−3^) for soils of the *Acacia gerrardii* (left) and the *Juniperus procera* (right) study sites in southwestern Saudi Arabia.

SOC content (g C kg^−1^) and SBD (g cm^−3^) exhibited significant inverse relationships, and this inverse relationship appeared stronger for the *J*. *procera* study site (r = -0.453), compared to the *A*. *gerrardii* study site (r = -0.225) (**Figure 6**), These inverse relationships were described by the following non-linear regression equations for the *A. gerradii* site, SBD = 2.001 + 1.177 exp^−0.054^× ^SOC content^ (*r* = −0.225), and for the *J. procera* site, SBD = 0.892 + 2.883 exp^–0.021 × SOC content^ (*r* = −0.453).

The two CCAs indicate that the positions of SBD, SOC content, SOC density, and SOC stock in the *A*. *gerradii* and J. *procera* ordinations were almost identical (**Appendix 3**): the first axis separated SOC density and SOC stock from SOC content and SBD and the second axis separated SBD from the three SOC parameters. For the *A. gerrardii* study site, none of the soil parameters had a statistically significant effect on SBD, SOC content, SOC density, or SOC stock (**Table 2**); although Axis 1 showed a negative relationship with silt content and clay content and a positive relationship with the other soil parameters, and Axis 2 revealed a negative relationship with pH, silt content, and clay content and a positive relationship with EC, TDS, and sand content (**Appendix 3**). For the *J*. *procera* study site, Axis 1 revealed a significant association between sand and clay and SBD, SOC content, SOC density, and SOC stock (*P* = 0.01) and the significance of silt was *P* = 0.05; the three other soil parameters were not significant (**Table 2**). Axis 1 of the *J*. *procera* ordination showed a negative association with pH, clay, silt, and EC and a positive association with TDS and sand, and Axis 2 revealed a negative association with clay, silt, EC, and TDs and a positive association with pH and sand (**Appendix 3**).

**Table 2:** Relationship (correlation) between soil parameters and soil bulk density (SBD), soil organic carbon (SOC) content, soil organic carbon (SOC) density, and soil organic carbon (SOC) stock at the (a) *Acacia gerrardii* and (b) *Juniperus procera* study sites in southwestern Saudi Arabia, detected using canonical correspondence analysis (CCA).

Soil parameters	*Acacia gerrardii*		*Juniperus procera*	
	Axis 1	Axis 2	Axis 1	Axis 2
pH	0.3588	-0.9334	-0.3669	0.9303
EC (mS cm^−1^)	0.3041	0.9526	-0.3711	-0.9286
TDS (ppt)	0.3284	0.9445	0.0456	-0.9990
Sand (%)	0.3634	0.9316	0.8294**	0.5587
Silt (%)	-0.1429	-0.9897	-0.7716*	-0.6361
Clay (%)	-0.5623	-0.8269	-0.8598**	-0.5106

EC, electrical conductivity; TDS, total dissolved solids; *P = 0.05; **P = 0.01, all other values are non-significant.

## 4 Discussion

Due to the important consequences SOC variation and dynamics around the globe (Walter et al., 2016; Shu et al., 2020; Yao et al., 2023), the results of an abundance of SOC studies have been published, and these results have been summarized in several recent reviews and meta-analyses (Beillouin et al., 2021, 2022, 2023; Devi, 2021). However, most of these published studies have been conducted in just five countries (Beillouin et al., 2021), and many areas around the globe are underrepresented. One such underrepresented area is the Arabian Peninsula and specifically Saudi Arabia. The current study assessing SOC in soils of *J*. *procera* and *A*. *gerrardi* highland forest study sites in southwestern Saudi Arabia is our effort to provide data for this understudied region.

### 4.1 Variation in SOC: elevation, climate, and species identity

Our results indicate that soils of the *J. procera* study site had significantly higher mean values for SOC content and SOC density compared to the mean values of the A. *gerrardii* study site (**Table 1**). And while the mean value of SOC stock at the *J*. *procera* study site was higher than that of the *A*. *gerrardii* study site, these differences were not statistically significant. Values of SOC content and SOC density generally decreased with increasing soil depth; although the highest values of SOC density for both the *P*. procera and *A*. *gerrardii* study sites occurred at lower soil depths, not at the upper-most soil layer (**Figure 5**, **Appendix 2**). Soil bulk density (SBD) increased with soil depth at both the *P*. *procera* and *A*. *gerrardii* study sites (**Figure 4**).

Comparison of our results for SOC content, SOC density, and SOC stock with previously published SOC studies conducted in Saudi Arabia indicate that salt marsh, coastal habitats, and mangrove forest, which are associated with high levels of plant productivity, have SOC levels that are generally higher than our results (El-Demerdash et al., 1995; Shaltout et al., 2020; Eid et al., 2016, 2019, 2023; Cusack et al., 2018; Al-Guwaiz et al., 2021). Conversely, our SOC results are similar to or higher than results reported for arid habitats with low productivity such as wadis (El-Sheikh et al., 2018; Osman et al., 2023) and a degraded highland forest site (Al-Ghamdi et al., 2021). Results of these three studies assessing SOC levels in arid habitats with low productivity in Saudi Arabia have soil parameters similar to those of our two study sites. In addition, our SOC results are generally consistent with the SOC values listed for tree species growing under similar soil conditions to those of our two study sies (see the review by Devi, 2021).

An assessment of the biotic and abiotic conditions at our two study sites points to several factors that contribute to the SOC results reported here: 1) the *J*. *procera* study site occurs at a significantly higher elevation than the *A*. *gerrardii* study site (means = 2775.5 m and 2235.7 m, respectively), 2) the *J*. *procera* study site experiences a more moderate climate regime compared to the *A*. gerrardii study site, 3) the identity of the two species that dominate the two study sites, 4) the value of SBD at the *J*. *procera* study site (1.62 g cm^−3^) was statistically lower compared to the *A*. *gerrardii* study site (2.30 g cm^−3^), and 5) the *P*. *procera* study site had a significantly higher clay content compared to the *A*. *gerrardii* study site (although the two study sites did not significantly differ for the other five soil parameters). Unfortunately, we did not conduct experiments that would allow us to partition the relative contributions of these various factors to the SOC variation reported here.

However, the distribution of tree species and vegetation types (i.e., vegetation cover) in arid and semi-arid regions such as southwestern Saudi Arabia, are influenced by elevation and, consequently, differences in temperatures and precipitation. Higher elevations experience lower temperatures, and more precipitation (a more moderate climate) compared to nearby lowland sites, and these conditions determine differences in the distribution of plant species, vegetation types, and plant biomass accumulation of highland and lowland habitats. In this region, *J*. *procera* is the most dominant component of vegetation at 2000 m a.s.l. and above (Chaudhary, 1999; El-Juhany, 2015). At 1000 m a.s.l. and below, where the climate is much hotter and drier, *J*. *procera* disappears and *A*. *gerrardii* dominates (El-Juhany, 2015). Difference in the SOC parameters we report here were determined, in part, by the joint effects of climate conditions and the identity of the tree species at the two study sites, as described by other authors (Hobbie et al., 2007; Díaz-Pinés et al., 2011; Angst et al., 2019; Yao et al., 2023).

### 4.2 Tree growth characteristics and SOC

Additionally, forest habitats typically have higher SOC levels compared to other habitats (e.g., grasslands and deserts) because forest vegetation has higher plant biomass accumulation and litterfall (see Devi, 2021). As plants grow, they add organic matter to the soil through decaying plant tissues, such as leaf litter, woody tissue, root tissues, and root exudates (Yao et al., 2023). Thus, we hypothesized that the tree species with growth characteristics associated with greater canopy size and volume and amounts of woody tissue would have the highest SOC values. Soils of the *J. procera* study site had higher levels for the three SOC parameters we measured, compared to the *A*. *gerrardii* study site and *J*. *procera* trees exhibited significantly higher values for DBH, tree radius, and basal area (*A*. *gerrardii* trees had significantly higher values for crown depth, crown diameter, and tree height, and crown volume and C/H ratio did not differ significantly for the two tree species). These results suggest that the higher SOC levels at the *J*. *procera* study site are associated with significant differences in woody tissue biomass (DBH and basal area) and tree radius, and the SOC levels of the *A*. *gerrardii* site were apparently not influenced by significantly different parameters associated with canopy size and canopy volume (crown depth, crown diameter, and tree height). This apparent lack of influence of canopy size and volume may stem from the fact that both trees are evergreen species (Elias, 1981; Farjon, 2005; El-Juhany, 2015), and lower foliage turnover rates may not make a significant contribution to the SOC levels at the two study sites. Our findings differ from the results reported by Hou et al. (2020), which showed that evergreen broadleaf trees, such as *A*. *gerrardii*, accumulate SOC at a faster rate compared to evergreen conifer trees. Future research will be needed to resolve this discrepancy.

### 4.3 SBD, soil texture, and SOC

Soil bulk density (SBD) is determined by soil composition (particle size and organic matter content), soil structure, moisture content, and soil compaction; and SBD influences several soil properties including porosity, available water content, hydraulic conductivity, and SOC levels (Wilson et al., 2009; Abdelbaki, 2018; Osman et al., 2023). Generally, the soil of habitats with abundant vegetation has lower SBD values compared to soil of habitats with sparse or no vegetation; and SBD is inversely related to SOC, i.e., low values of SBD are often associated with higher values of SOC (Périé and Ouimet, 2008; Zhao et al., 2015). Our results are consistent with this pattern, SBD was significantly lower at the *J*. *procera* study site compared to the *A*. *gerrardii* study site, and SOC content and SOC density was significantly higher at the *J*. *procera* site (**Table 1**).

Differences in SOC levels we detected at the *J*. *procera* and *A*. *gerrardii* study sites are also significantly associated with soil texture (i.e., clay content) (**Figure 3**). Clay content was significantly higher at the *J*. *procera* study site compared to the A. *gerrardii* study site, and the *J*. *procera* site had significantly higher mean values for SOC content and SOC density and a higher mean value of SOC stock compared to the *A*. *gerrardii* site (**Table 1**). This association occurred because soils with high amounts of clay, which have larger surface areas and poor drainage, can store higher amounts of SOC and protect this SOC through aggregation and absorption mechanisms, compared to sandy soils (Hassink, 1997; Chenchouni and Neffar, 2022; Osman et al., 2023).

### 4.4 Limitations of the study: leaf litter and understory vegetation

In this study, we analyzed SOC, tree growth characteristics and soil parameters in two study areas, one higher elevation site dominated by *J*. *procera* and one lower elevation site dominated the *A*. *gerrardii*. Consequently, this experiment design does not include any replication for the two forest study sites; thus, this design is an example of pseudoreplication (*sensu* Hurlbert, 1984). While pseudoreplication was unavoidable in this study due to the large number of tree growth parameters and the large amount of soil samples we analyzed, future research should analyze addition stands of both three species to better assess variation in the SOC results we report here. In addition, future research should conduct analyses to more precisely partition the relative contributions of the various factors we examined in determining the SOC variation reported here.

The amount and chemical composition of leaf litter (and woody tissue and roots) influences SOC levels in several ways: higher amounts of leaf litter and other tissues increase SOC; high lignan content of litter reduces decomposition rates; nutrient-rich litter decomposes faster and releases more carbon into the soil than nutrient-poor litter; litter chemistry affects microbial communities in the soil; and the chemistry of litter interacts with soil parameters such as pH and soil texture, which can enhance or reduce SOC levels (Erhagen et al., 2013; Chen et al., 2015; Capellesso et al., 2016) (Liang et al., 2024). Unfortunately, we did not quantify the amount of litterfall of the two tree species at the two study sites; we do not know how often litterfall occurs, and we do not know the decomposition rates of this litter. However, both *J*. *procera* and *A*. *gerrardii* are evergreen tree species (Aref et al., 1995; Chaudhary, 1999; El-Juhany, 2015); thus litterfall probably occurs on a continuous basis as older needles and leaves senesce. In addition, we did not determine the chemical compsition of the litter for the two study species, and we did not determine how these chemicals influence SOC. Assessing the role of leaf litter on the SOC variation we detected for these two trees should be a focus of future research.

The abundance and diversity of the understory vegetation influence SOC variation. More abundant and diverse understory vegetation is associated with lower SBD values and higher SOC levels compared to those with sparse or no vegetation (Périé and Ouimet, 2008; Zhao et al., 2015). **Figures 1a, b** contain photos of the two study sites, and these photos reveal more abundant and diverse understory vegetation at the *J*. *procera* study sites, which had higher SOC levels compared to the *A*. *gerradii* sites. Understory vegetation influences SOC levels in much the same way leaf litter does: 1) understory plants enhance SOC by contributing the organic matter of litter, roots, and decaying plant material, 2) understory vegetation increases SOC by promoting microbial diversity and activity, which enhances the decomposition of organic matter, and 3) the roots of understory vegetation improves soil structure, and increases water retention and the availability of nutrients, thereby increasing SOC levels (Pan et al., 2018; Deng et al., 2023; Liang et al., 2024). Therefore, we recommend that future studies should sample the understory vegetation at these two study sites to better determine the role of understory vegetation on the SOC variation reported here.

## 5 Conclusion

Most published SOC studies have been conducted in just five countries, and many areas around the globe are underrepresented in the literature of this research topic. To the best of our knowledge this is the first study to assess the SOC variation of highland forests in southwestern Saudi Arabia. Our results indicate that values of SOC content, SOC density, and SOC stock are higher for the *J*. *procera* study site compared to the *A*. *gerrardii* study site. These outcomes appear to be influenced by differences in elevation, climate, species identity, clay content, and soil bulk density. In addition, higher SOC levels at the *J*. *procera* study site appear to be associated with significant differences in woody tissue biomass (DBH and basal area) and tree radius, and not parameters associated with canopy size and canopy volume. Yet to be determined, are the roles of litterfall and the understory vegetation in contributing to the SOC variation of these two forest communities.

The high elevation sites where *J*. *procera* dominates contain higher plant species diversity and productivity compared to other regions in Saudi Arabia. Unfortunately, *J*. *procera* is listed as a threatened species on the IUCN Red List due to overexploitation. *Juniperus procera* and *A*. *gerrardi* are the dominant species in the plant communities they occur in; thus, these species and communities should be the focus of effective management actions because they play an important role in carbon sequestration and are also important for biodiversity conservation and the maintenance of ecosystem function. We recommend that overexploitation of *J*. *procera* should be minimized (or even curtailed), as should the overharvesting of both tree species and any activities that disturb the soil profile in these plant communities. Finally, efforts should be made to restore these two tree species and plant communities in areas where they have been eliminated or altered.

## Data Availability

The original contributions presented in the study are included in the article/**Supplementary Material**. Further inquiries can be directed to the corresponding authors.
