# Supplementary material for: Tree growth characteristics, soil parameters, and soil organic carbon of highland Juniper and Acacia forests in Southwestern Saudi Arabia
Source: Front Plant Sci. 2026 Jan 30;16:1693276. doi: 10.3389/fpls.2025.1693276 (PMC12901478; doi:10.3389/fpls.2025.1693276)
Supplement: Supplementary file 1 [file Table1.docx]

Supplementary Material

## Supplementary Figures


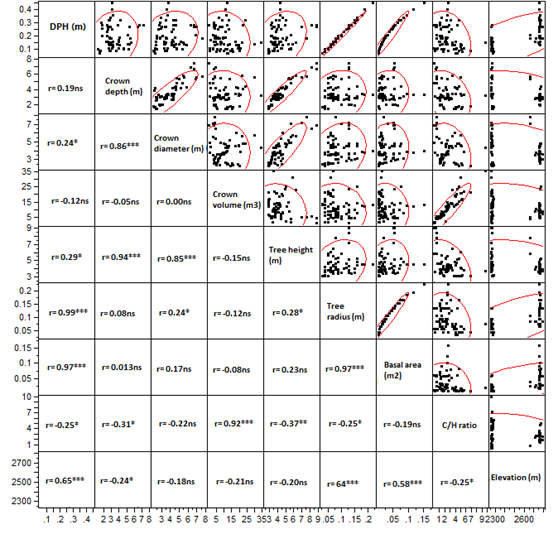


**Supplementary Figure 1.** Scatter matrix of correlation coefficients of elevation and the eight *Juniper procera* and *Acacia gerardias* tree growth characteristics we measured at the two study sites in southwestern Saudi Arabia, as well as the correlation between the eight tree growth characteristics.


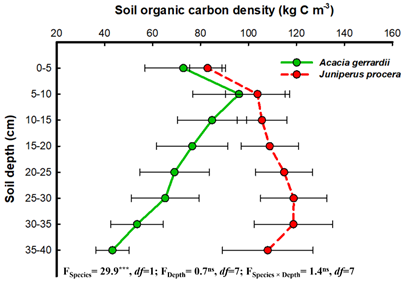


**Supplementary Figure 2.** Mean soil organic carbon density (kg C m^−3^) values at eight soil depth (cm) for the *A. gerrardii* and *J. procera* study sites in southwestern Saudi Arabia. Horizontal bars indicate the standard errors of the means. *F*-values represent the two-way ANOVAs. Depths: 0 – 5, 5 –10, 10 –15, 15 – 20, 20 – 25, 25 – 30, 30 – 35, 35 – 40 cm. According to Tukey’s range test, soil organic carbon density values do differ significantly with soil depth.


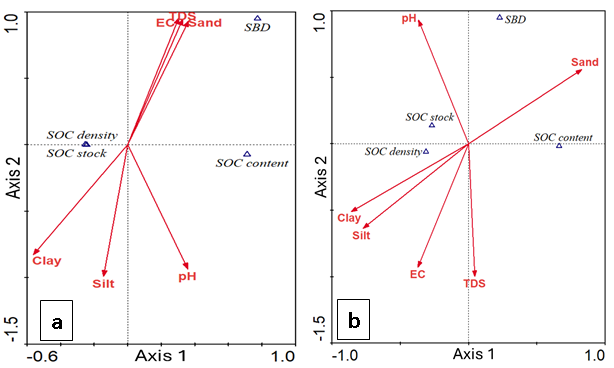


**Supplementary Figure 2.** Canonical correspondence analysis (CCA) diagrams showing the relationship between the six soil parameters and soil bulk density (SBD), soil organic carbon (SOC) content, soil organic carbon (SOC) density, and soil organic carbon (SOC) stock in **(a)** *A. gerrardii* and **(b)** *J. procera* study sites in southwestern Saudi Arabia.
